# Mutational analysis of the transmembrane α4-helix of *Bacillus thuringiensis* mosquito-larvicidal Cry4Aa toxin

**DOI:** 10.1007/s00284-023-03602-8

**Published:** 2024-01-28

**Authors:** Hirokazu Takahashi, Mami Asakura, Toru Ide, Tohru Hayakawa

**Affiliations:** Graduate School of Interdisciplinary Science and Engineering in Health Systems, Okayama University, 3-1-1 Tsushima-Naka, Kita-Ku, Okayama 700-8530 Japan

## Abstract

Cry4Aa, produced by *Bacillus thuringiensis* subsp. *israelensis*, exhibits specific toxicity to larvae of medically important mosquito genera. Cry4Aa functions as a pore-forming toxin, and a helical hairpin (α4-loop-α5) of domain I is believed to be the transmembrane domain that forms toxin pores. Pore formation is considered to be a central mode of Cry4Aa action, but the relationship between pore formation and toxicity is poorly understood. In the present study, we constructed Cry4Aa mutants in which each polar amino acid residues within the transmembrane α4 helix was replaced with glutamic acid. Bioassays using *Culex pipiens* mosquito larvae and subsequent ion permeability measurements using symmetric KCl solution revealed an apparent correlation between toxicity and toxin pore conductance for most of the Cry4Aa mutants. In contrast, the Cry4Aa mutant H178E was a clear exception, almost losing its toxicity but still exhibiting a moderately high conductivity of about 60% of the wild-type. Furthermore, the conductance of the pore formed by the N190E mutant (about 50% of the wild-type) was close to that of H178E, but the toxicity was significantly higher than that of H178E. Ion selectivity measurements using asymmetric KCl solution revealed a significant decrease in cation selectivity of toxin pores formed by H178E compared to N190E. Our data suggest that the toxicity of Cry4Aa is primarily pore related. The formation of toxin pores that are highly ion-permeable and also highly cation-selective may enhance the influx of cations and water into the target cell, thereby facilitating the eventual death of mosquito larvae.

## Introduction

The gram-positive, spore-forming bacterium *Bacillus thuringiensis* subsp. *israelensis* (Bti) exhibits strong toxicity against larvae of medically important mosquito genera, such as *Aedes*, *Anopheles*, and *Culex* [1, 2], and it has therefore been used as a mosquito-control agent for many years [3]. The mosquito-larvicidal activity of Bti resides in three Cry toxins (Cry4Aa, Cry4Ba, and Cry11Aa) and a Cyt toxin (Cyt1Aa) [4, 5]. Cry toxins exhibit potent toxicity against mosquito larvae. Although the mosquito-larvicidal activity of Cyt1Aa is very low, it synergizes with other Cry toxins [6–9]. Synergistic toxicity has also been observed between Bti Cry toxins [2, 10], which is advantageous for the use of Bti as a mosquito-control agent. However, the mechanism of the synergistic toxicity is poorly understood.

Three-dimensional structure models of Cry4Aa, Cry4Ba, and Cry11Aa constructed using X-ray crystallography data indicate that these toxins share a similar three-domain architecture (domains I, II, and III) [11–13]. In general, domain I consists of a bundle of seven amphipathic α-helices at the N-terminus, and this domain is thought to form a transmembrane pore. Domain II, which consists of three antiparallel β-sheets, is a receptor-binding domain. Loops exposed on the surface of the toxin molecule are thought to function as the receptor-binding site. Domain III consists of antiparallel β-sheets that form a β-sandwich fold with a jellyroll topology, and this domain is assumed to be involved in controlling the structural integrity and/or receptor binding of the toxin [4]. According to the colloid-osmotic lysis model, after interaction with specific receptors, three-domain Cry toxins form pores in the target cell membrane. The toxin-produced pores allow the influx of water along with ions, leading to swelling and eventual lysis of target cells [14].

Research indicates that Cry4Aa forms cation-selective channel pores in planar lipid bilayers, and the ion selectivity of these channel pores seems to be correlated with the toxicity of Cry4Aa [15]. To further characterize the relationship between channel pore ion selectivity and toxicity, we constructed a series of mutants in which each polar amino acid residues within the transmembrane α4 helix of domain I was replaced with glutamic acid. The Cry4Aa mutants were subjected to bioassays using *C. pipiens* mosquito larvae and ion-permeability measurements using planar lipid bilayers.

## Materials and Methods

### Construction of Cry4Aa Mutants

**Fig. 1:**
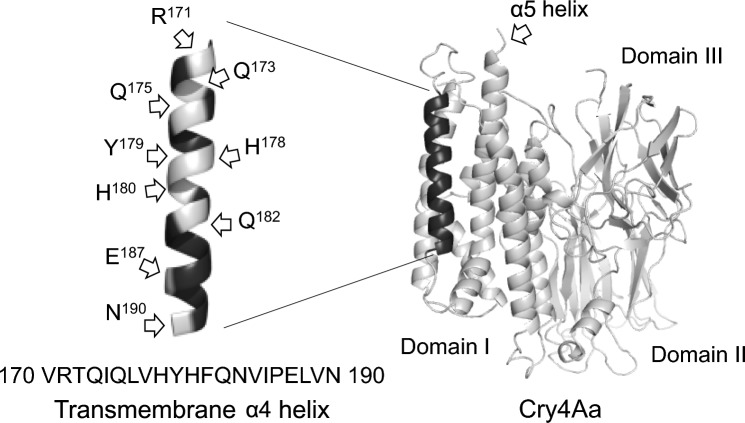
Structures of the Cry4Aa active toxin and transmembrane α4 helix. Positions of polar amino acid residues in the α4 helix are indicated. The three-dimensional structure was generated with PyMOL software [18] using the Cry4Aa PDB code (2c9k)

In the structural model of Cry4Aa [12], the transmembrane α4 helix is composed of 21 amino acid residues, 11 of which (R^171^, T^172^, Q^173^, Q^175^, H^178^, Y^179^, H^180^, Q^182^, N^183^, E^187^, and N^190^) are residues with a polar side chain (Fig. 1). In the present study, we constructed a series of Cry4Aa mutants in which polar amino acid residues (except E^187^) in the α4 helix was individually replaced with glutamic acid. As glutamic acid has a negatively charged bulky side chain, the replacement was expected to cause a significant change in both ion permeability through the toxin pores and resultant mosquito-larvicidal activity. Mutations were introduced via site-directed mutagenesis as described previously [16]. The expression vector pGST-Cry4Aa-S1 [17] was used as a template. The primers used for mutagenesis are listed in Table 1. The introduction of each desired mutation was confirmed by DNA sequencing.

**Table 1:** Nucleotide sequences of primers used for site-directed mutagenesis

Primer	Primer sequence (5′ → 3′)	Mutant
4Aa-171E-f	GAAACCCAAATCCAACTGGTGCACT	R171E
4Aa-171-175-r	CACGTCTTGGGTGTTTTGCGGGTT	
4Aa-173E-f	CGTACCGAAATCCAACTGGTGCACT	Q173E
4Aa-171-175-r		
4Aa-175E-f	CGTACCCAAATCGAACTGGTGCACT	Q175E
4Aa-171-175-r		
4Aa-178E-f	GAATACCACTTCCAAAACGTCATC	H178E
4Aa-178-180-r	CACCAGTTGGATTTGGGTACGCAC	
4Aa-179E-f	CACGAACACTTCCAAAACGTCATC	Y179E
4Aa-178-180-r		
4Aa-180E-f	CACTACGAATTCCAAAACGTCATC	H180E
4Aa-178-180-r		
4Aa-182E-f	GAAAACGTCATCCCGGAACTGGTG	Q182E
4Aa-182-183-r	GAAGTGGTAGTGCACCAGTTGGAT	
4Aa-190E-f	GAAAGCTGCCCGCCGAACCCGAGC	N190E
4Aa-190-r	CACCAGTTCCGGGATGACGTTTTG	

### Preparation of Recombinant Cry4Aa

Cry4Aa mutants were expressed as glutathione *S*-transferase (GST) fusion protein in *Escherichia coli* BL21 and purified as described previously [17]. Briefly, *E. coli* cells harboring the expression vector were cultured in Terrific Broth containing ampicillin (100 µg/mL) at 37 °C until the OD_600_ reached 0.5–0.7. Expression of GST-Cry4Aa was induced by incubating the cells in medium containing 0.1 mM isopropyl-β-d-thiogalactopyranoside at 20 °C for 4 h. Upon disruption of the *E. coli* cells by sonication, GST-Cry4Aa was purified using glutathione-Sepharose 4B resin (GE Healthcare Bio-Sciences AB, Uppsala, Sweden) according to the manufacturer’s instructions.

For electrophysiologic analyses, the active toxin fragment (G^58^–Q^695^) of Cry4Aa was cleaved from GST-Cry4Aa bound to the resin using thrombin (Cytiva, Tokyo, Japan) according to the manufacturer’s instructions. The protein concentration was estimated using a protein assay dye regent (Bio-Rad Laboratories, Inc., Hercules, CA) with bovine serum albumin as the standard. Purified proteins were analyzed by sodium dodecyl sulfate–polyacrylamide gel electrophoresis (SDS-PAGE), followed by visualization of protein bands using Coomassie brilliant blue regent (CBB stain one, Nacalai Tesque, Inc., Kyoto, Japan).

### Trypsin Treatment of Cry4Aa Mutants

In general, Cry toxins are activated by trypsin-like proteases in the midgut juice of susceptible insect larva, generating a protease-resistant active toxin fragment. Trypsin treatment is thus thought to serve as a presumptive test of the folding fidelity of Cry toxins [19]. In the present study, 1 μg of purified GST-Cry4Aa was treated with 20 ng of trypsin for 2 h at 37 °C in 100 mM Tris–HCl (pH 8.0). The digests were then analyzed by SDS-PAGE.

### Measurement of Mosquito-Larvicidal Activity

The mosquito-larvicidal activity of Cry4Aa mutants was analyzed by bioassays using *C. pipiens* mosquito larvae (3rd instar). Mosquito larvae were reared from eggs that were kindly supplied by the Research and Development Laboratory, Dainihon Jochugiku Co., Ltd. (Osaka, Japan). Briefly, 40 μg of purified GST-Cry4Aa was adsorbed onto 2 mg of latex beads (0.8 μm diameter, Sigma-Aldrich Corp., St. Louis, MO) for 1 h at room temperature and then administered to the mosquito larvae as a diet. Bioassays were carried out in a 96-well microtiter plate with a single larva per well and 48 larvae per each concentration in an assay. Mortality was recorded 48 h after toxin administration, and the 50% lethal dose (LC_50_) was determined using PROBIT analysis [20]. All bioassay experiments were repeated three times. Statistical significance was evaluated using Student’s *t* test.

### Electrophysiological Analysis

Ion permeability of the channel pores formed by Cry4Aa was analyzed using an electrophysiologic procedure. The experimental apparatus consisted of two chambers (upper, *cis* chamber; lower, *trans* chamber), such that the voltage in the solution of the *cis* chamber was connected to a patch-clamp amplifier by a Ag/AgCl electrode-defined membrane potential. The micropipette used as the *cis* chamber was constructed from a glass capillary (GD-1.2, Narishige Scientific Instrument Lab., Tokyo, Japan) using a P-97 Sutter Instruments puller (Novato, CA) and then heat-polished to a tip diameter of 5–10 μm. The micropipette (*cis* chamber) was filled with solution containing Cry4Aa active toxin at a concentration of 5 μg/mL, and a solvent-free lipid bilayer was formed at the tip of the micropipette according to the Tip-Dip method [21]. Briefly, the tip of the micropipette was inserted into the solution in a 0.5-mL microtube (*trans* chamber), and 100 μL of phosphatidylcholine solution (5 mg/mL in hexane) was carefully added to the surface of the solution in the *trans* chamber. The hexane was quickly evaporated to form a phospholipid monolayer, and then the lipid bilayer was formed by repetitive dipping of the pipette tip into the solution. Cry4Aa was incorporated into the lipid bilayer while applying a 20-mV holding potential across the lipid bilayer. Data were analyzed using pClamp software (Molecular Devices LLC, San Jose, CA).

Currents through the channel pore formed by wild-type or mutant Cry4Aa were recorded in a symmetrical solution containing 150 mM KCl and 10 mM Tris–HCl (pH 8.0). Conductance of the channel pore was determined from the slope of the current–voltage relationship plotted between the observed current and the corresponding applied voltages. In contrast, to analyze the anion-cation selectivity of the channel pores, currents through the channel pore were recorded in the presence of a four fold gradient of KCl across the lipid bilayer (600 mM KCl and 10 mM Tris–HCl [pH 8.0] in the *cis* chamber, 150 mM KCl and 10 mM Tris–HCl [pH 8.0] in the *trans* chamber). The zero-current reversal potential (*V*_R_) was obtained as the X-intercept of the current–voltage relationship line. The *V*_R_ values were then corrected by the values of the junction potential. The junction potential was determined as − 0.4 mV under this condition [15]. The cation–anion permeability ratio (*P*_K_/*P*_Cl_) was calculated using the Goldman-Hodgkin-Katz equation with the *V*_R_, as reported previously [22]. Statistical significance was evaluated using Student’s *t* test.

## Results

### Construction of Cry4Aa Mutants

**Fig. 2:**
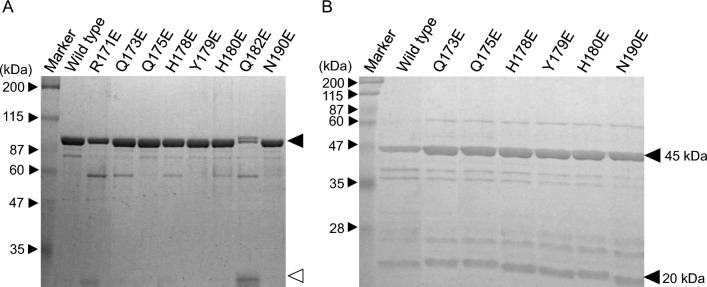
Recombinant wild-type Cry4Aa and mutant toxins. **A** The GST-Cry4Aa wild-type and mutant toxins were purified using glutathione beads. Purified proteins were analyzed by 10% SDS-PAGE. **B** The GST-Cry4Aa wild-type and mutant toxins were treated with trypsin. Proteins were analyzed by 15% SDS-PAGE

Eight Cry4Aa mutants (R171E, Q173E, Q175E, H178E, Y179E, H180E, Q182E, and N190E) in which polar amino acid residues within the transmembrane α4 helix were individually replaced with a glutamic acid residue were constructed. Most of the Cry4Aa mutants were successfully expressed in *E. coli*, and no differences in size or expression level were observed between the wild-type and mutant toxins (Fig. 2A). However, the Cry4Aa mutants R171E and Q182E seemed to be unstable, as a degraded polypeptide band was detected at approximately 30 kDa by SDS-PAGE (Fig. 2A). The Cry4Aa mutants R171E and Q182E were therefore not used for further investigation. As glutamic acid possesses a negatively charged bulky side chain, the replacement at positions R^171^ and Q^182^ may have affected the integrity of the Cry4Aa structure, leading to degradation.

The remaining six mutants (Q173E, Q175E, H178E, Y179E, H180E, and N190E) were subjected to trypsin treatment. In general, Cry4Aa is synthesized as a 130-kDa protoxin that is activated by larval trypsin-like gut proteases that cleave the toxin into two protease-resistant fragments of 20 and 45 kDa via intramolecular cleavage of a 60-kDa intermediate [23]. SDS-PAGE analysis revealed protease-resistant fragments with molecular masses of 20 and 45 kDa for all mutants, as well as for wild-type Cry4Aa (Fig. 2B). This suggested that the remaining six Cry4Aa mutants retained folding fidelity compared with the wild-type toxin, and may reach target site (brush border membrane of the midgut epithelium) passing through protease-rich midgut juice.

### Mosquito-Larvicidal Activity of Cry4Aa Mutants

**Fig. 3:**
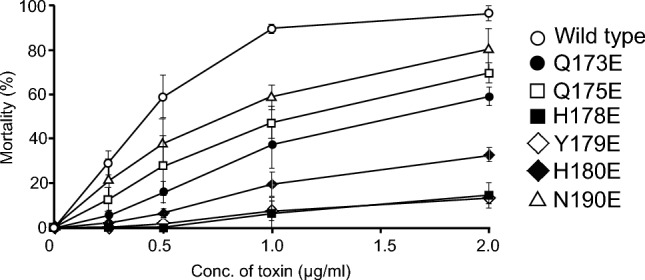
Mosquito-larvicidal activity of Cry4Aa wild-type and mutant toxins. Mortality of *C. pipiens* larvae was recorded 48 h after toxin administration. The bioassays were repeated three times. Data are shown as the mean and standard deviation

Purified GST-Cry4Aa was administered to *C. pipiens* larvae, and the LC_50_ values were calculated based on the mortality of larvae at 48 h after administration. Wild-type Cry4Aa was highly toxic against *C. pipiens* larvae, with an LC_50_ value (95% confidence limits) of 0.40 (0.37–0.42) μg/mL (Fig. 3); by contrast, GST used as a negative control exhibited no toxicity at concentrations up to 2 μg/mL (data not shown). The Cry4Aa mutants exhibited decreased toxicity compared to the wild-type toxin, but the degree of the decrease varied with the position of the mutation in the α4 helix (Fig. 3). For example, mutants Q173E, Q175E, and N190E, which harbored the mutation in the terminal region of the α4 helix, exhibited a relatively limited decrease in toxicity, and their LC_50_ values (95% confidence limits) could be determined as 1.51 (1.38–1.66), 1.07 (0.99–1.16), and 0.73 (0.68–0.79) μg/mL, respectively (Fig. 3). In contrast, mutants H178E, Y179E, and H180E, which harbored the mutation in the internal region of the α4 helix, exhibited significantly decreased toxicity, with LC_50_ values over 2 μg/mL (Fig. 3). When statistical significance was considered at *P* < 0.05, the order of mosquito-larvicidal activity determined based on LC_50_ values was WT > N190E > Q173E ≈ Q175E > (H178E, Y179E, and H180).

### Ion Permeability of the Channel Pores Formed by Cry4Aa Mutants

**Fig. 4:**
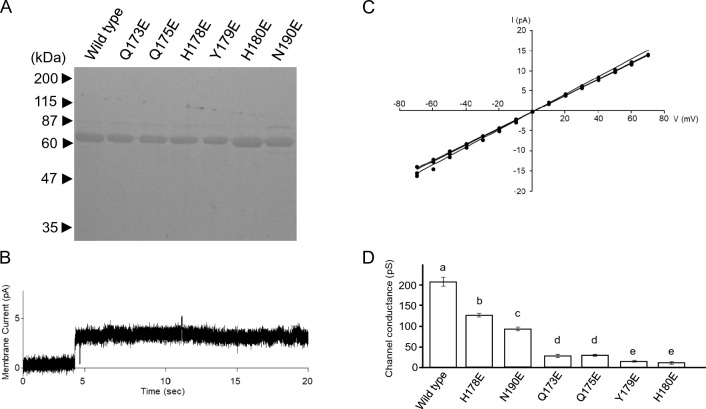
Electrophysiological analysis of Cry4Aa wild-type and mutant toxins. **A** Active polypeptides of Cry4Aa wild-type and mutant toxins. Active toxin polypeptides were analyzed by 10% SDS-PAGE. **B** Typical current trace recorded when a Cry4Aa channel-pore was formed in an artificial lipid bilayer. Formation of Cry4Aa channel pores was facilitated applying a 20-mV holding potential across the lipid bilayer. **C** Current–voltage relationship of wild-type Cry4Aa channel pores. The channel-currents recorded in symmetrical 150 mM KCl solutions were plotted versus applied voltage. The experiment was repeated 3 times independently. **D** Single channel conductance of the channel pores formed by Cry4Aa wild-type and mutant toxins. The channel conductance was determined from the slope of the current–voltage relationship. The mean and standard deviation of the values are shown as a bar graph. Different alphabet indicates that the difference is statistically significant (*P* < 0.05) using Student’s *t* test

Cry4Aa active toxins were cleaved from GST-Cry4Aa bound to the resin using thrombin. SDS-PAGE analysis revealed a 70-kDa polypeptide for both the wild-type and mutant Cry4Aa toxins (Fig. 4A). The 70-kDa polypeptide was very similar in size to the Cry4Aa active toxin reported previously (Fig. 4A) [15].

The ion permeability of the channel pores of the 70-kDa active toxins was analyzed under symmetrical buffer (150 mM KCl, 10 mM Tris–HCl [pH 8.0]) conditions. Upon preparation of the solvent-free planar lipid bilayer, formation of the toxin channel pores was facilitated by applying voltage (20 mV). At approximately 30 min after initiation, a sharp increase in membrane current was usually observed (Fig. 4B). This was thought to be indicative of current flowing through the Cry4Aa channel pores formed in the planar lipid bilayer, as no similar current transition was observed in the recording for the control lacking Cry4Aa. The Cry4Aa channel pores formed in the solvent-free planar lipid bilayer seemed to remain in a stable open state for at least several minutes, and the currents were recorded between − 70 and + 70 mV. The currents were plotted versus the corresponding applied voltage to generate current–voltage relationships. Recordings were repeated three times using independently prepared samples of 70-kDa Cry4Aa active toxin. The current–voltage relationships for the channel pores formed by wild-type Cry4Aa were linear and exhibited a similar conductance level in each measurement (Fig. 4C). The single-channel conductance of the wild-type Cry4Aa channel pores was thus 208 ± 10 pS in symmetrical buffer containing 150 mM KCl. The value was similar to that (187 ± 10 pS) reported previously [15].

The channel pores formed by Cry4Aa mutants were analyzed in comparison with those formed by wild-type Cry4Aa. The current–voltage relationships for the mutant channel pores were linear, but the slopes were significantly shallower than that of the wild-type toxin. Indeed, the single-channel conductance for the channel pores formed by the Cry4Aa mutants Q173E, Q175E, H178E, Y179E, H180E, and N190E were 28 ± 4, 30 ± 2, 127 ± 4, 16 ± 2, 12 ± 3, and 93 ± 5 pS, respectively (Fig. 4D). When statistical significance was considered at *P* < 0.05, the order of the single-channel conductance was WT > H178E > N190E > Q173E ≈ Q175E > Y179E ≈ H180E.

Interestingly, when this result was compared with the toxicity of Cry4Aa mutants (Fig. 3), an apparent correlation was observed between the single-channel conductance and the toxicity of most of the mutants. This suggested that the conductance of the channel pores plays an important role in the mosquito-larvicidal activity of Cry4Aa. In contrast, Cry4Aa mutant H178E was an obvious exception. Despite of significant reduction in mosquito-larvicidal activity (Fig. 3), the channel pores formed by mutant H178 showed significantly higher conductance than those of other mutants (Fig. 4D). This suggested that other factors in addition to the conductance of the channel pores are important for mosquito-larvicidal activity.

### Anion-Cation Selectivity of the Channel Pores Formed by Cry4Aa Mutants

To further characterize Cry4Aa mutant H178E, the anion-cation selectivity of the channel pores was analyzed in the presence of asymmetric buffer and a four fold gradient of KCl across the lipid bilayer (600 mM KCl and 10 mM Tris–HCl [pH 8.0] in the *cis* chamber, 150 mM KCl and 10 mM Tris–HCl [pH 8.0] in the *trans* chamber). Cry4Aa mutant N190E was selected for comparison, as the channel pores formed by this mutant showed a channel conductance of 93 ± 5 pS, which was relatively close to that of mutant H178E (127 ± 4 pS), but the toxicity was significantly greater than that of mutant H178E (Figs. 3, 4D). Recordings were repeated three times using independently prepared samples of 70-kDa active toxin.

**Fig. 5:**
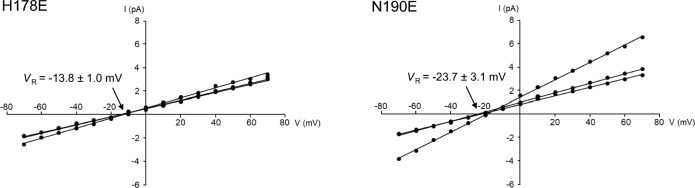
Anion-cation selectivity of the channel pores formed by Cry4Aa mutants H178E and N190E. Channel currents were recorded with a four fold gradient of KCl across the lipid bilayer. The experiment was independently repeated three times. The mean (standard deviation) *V*_R_ was determined using each fitted line

The *V*_R_ value for the channel-pores formed by mutant N190E was − 23.7 ± 3.1 mV, with a calculated *P*_K_/*P*_Cl_ permeability ratio of 6.10 (Fig. 5). This was similar to or somewhat higher than the previously reported value for wild-type Cry4Aa (4.9) [15]. In contrast, the *V*_R_ value for the channel-pores formed by mutant H178E was − 13.8 ± 1.0 mV, with a calculated *P*_K_/*P*_Cl_ permeability ratio of 2.55 (Fig. 5). This was significantly lower (*P* < 0.05) than that of mutant N190E, suggesting that this decrease was one of the causes of the decrease in mosquito-larvicidal activity.

## Discussion

According to the widely accepted pore-formation model for insecticidal Cry toxins (umbrella model), Cry4Aa is thought to insert a helical hairpin (α4-loop-α5) of domain I into the target cell membrane to form toxin pores. In particular, the hydrophilic face of the α4 helix is thought to line the pore lumen and participate in ion conduction [24, 25]. Therefore, to investigate the relationship between the ion-permeability of toxin pores and toxicity, we constructed Cry4Aa mutants in which each polar amino acid residues in the α4 helix was replaced with glutamic acid (Fig. 1). As glutamic acid has a negatively charged bulky side chain, the replacement was expected to cause a significant change in both ion permeability through the toxin pores and resultant mosquito-larvicidal activity.

In the present study, we prepared six Cry4Aa mutants, Q173E, Q175E, H178E, Y179E, H180E, and N190E. Bioassays using *C. pipiens* mosquito larvae revealed a significant decrease in the mosquito-larvicidal activity of all six mutants (Fig. 3). Similarly, ion permeability measurements revealed a significant decrease in the conductance of channel pores for all six mutants (Fig. 4D). Because an apparent correlation was observed between the mosquito-larvicidal activity and conductance of all Cry4Aa mutants except H178E (Figs. 3, 4D), conductance was thought to be a determinant of Cry4Aa mosquito-larvicidal activity. Similar observations have been reported for another mosquito-larvicidal toxin, Cry4Ba. Significant decreases were observed in both toxicity and conductance of the toxin pores for mutants N166I and N166A, in which N^166^ in Cry4Ba transmembrane α4-α5 was replaced with isoleucine (I) and alanine (A), respectively [26].

On the other hand, H178E was a clear exception among the Cry4Aa mutants constructed in this study. The toxicity of H178E (LC_50_ > 2 μg/mL) was significantly weaker than that of N190E (LC_50_ = 0.73 μg/mL) (Fig. 3), but the conductance of the toxin pores formed by H178E (127 pS) was apparently higher than that of N190E (93 pS) (Fig. 4D). To identify other factors that affect mosquito-larvicidal activity in addition to conductance, we compared the *P*_K_/*P*_Cl_ permeability ratio of channel pores between H178E and N190E. The *P*_K_/*P*_Cl_ permeability ratio for pores formed by H178E was 2.55, which was significantly lower than that of N190E (6.10). In our earlier research using the identical device and method, we determined that the wild-type Cry4Aa channel pores had a *P*_K_/*P*_Cl_ permeability ratio of 4.9 [15]. Considered together with this observation, our result suggested that the channel pore cation selectivity was another factor that affect mosquito-larvicidal activity in addition to conductance. Similar observations have been reported in mutational analyses targeting the transmembrane region of the mosquito-larvicidal Mpp46Ab toxin [27, 28]. Mpp46Ab (formerly designated Cry46Ab), a mosquito-larvicidal toxin with an aerolysin-type architecture, is structurally quite different from Cry4Aa, but like Cry4Aa, it has been shown to act as a pore-forming toxin [29].

Two models, known as “the pore formation model” and “the cell signaling transduction model”, have been proposed to explain the insecticidal mechanism of Cry toxins. According to the pore formation model, after interaction with specific receptors, the Cry toxin forms pores in the target cell membrane. The toxin pores allow the influx of water along with ions, leading to swelling and eventual lysis of the target cells [14]. By comparison, the cell signaling transduction model describes the interaction between Cry toxins and cadherin receptors, which induce activation of the G protein receptor and adenylyl cyclase, in turn triggering a Mg^2+^-dependent intracellular signaling pathway. Increased cAMP levels activate protein kinase A, which triggers a cascade of physiologic processes that ultimately induce programmed death via lysis [30]. Taken together with our observations in this study, the toxicity of Cry4Aa against *C. pipiens* mosquito larvae appears to primarily be due to pore formation. The formation of channel pores with high ion permeability and high cation selectivity may facilitate the influx of cations and water into the cell, thereby disrupting the membrane potential and causing cell swelling and lysis and the eventual death of the host larva. In addition, we observed that replacement of H^178^ with glutamic acid (H178E) caused a significant decrease in the *P*_K_/*P*_Cl_ permeability ratio of the channel pores, suggesting that the H^178^ residue is directly/indirectly involved in determining ion permeability through Cry4Aa pores. It would be of interest to investigate the role of the H^178^ residue in ion permeability through Cry4Aa pores and the resultant effect on toxicity using more-comprehensive mutational analyses.

## Conclusion

In the present study, to investigate the relationship between pore formation and toxicity in the mosquito-larvicidal Cry4Aa, we constructed a series of mutants in which each polar amino acid residue within the transmembrane α4 helix was replaced by glutamic acid. Bioassay with *C. pipiens* mosquito larvae and subsequent ion permeability measurements using artificial lipid bilayers revealed an apparent correlation between toxicity and toxin pore conductance for most of the Cry4Aa mutants. In addition, ion selectivity measurements using asymmetric KCl solution of the H178E and N190E mutants suggested that the decrease in cation selectivity of the toxin pores was one of the causes of the decrease in mosquito-larvicidal activity. Our data suggest that Cry4Aa toxicity is primarily related to pore characteristics. The formation of toxin pores that are highly ion-permeable and also highly cation-selective may enhance the influx of cations and water into the target cell, thereby facilitating membrane potential disruption, cell swelling, cell lysis, and the eventual death of mosquito larvae.

## Data Availability

Data are available from the authors upon reasonable request.
